# Decoding an Uncommon Form of Inflammatory Polyposis Colitis

**DOI:** 10.7759/cureus.78157

**Published:** 2025-01-28

**Authors:** Nargiz Gasimova, Daria Yunina-Distefano

**Affiliations:** 1 Department of Internal Medicine, Overlook Medical Center, Atlantic Health System, Summit, USA; 2 Department of Medicine, Division of Gastroenterology, Overlook Medical Center, Atlantic Health System, Summit, USA

**Keywords:** blood protein anomalies, chronic diarrhea, enteropathy, gastrointestinal polyposis, hypoalbuminemia, inflammatory polyposis colitis, malnutrition, metabolic disorders, weight loss

## Abstract

Inflammatory polyposis colitis is a rare, non-hereditary gastrointestinal disorder characterized by chronic diarrhea, diffuse gastrointestinal polyposis, and systemic features such as hypoalbuminemia, weight loss, and dermatological changes. The etiology remains unknown, with diagnosis reliant on clinical, endoscopic, and histopathological findings. This case describes a 79-year-old male with inflammatory polyposis colitis who responded to corticosteroid therapy. The report emphasizes the diagnostic and therapeutic challenges of this condition.

## Introduction

Inflammatory polyposis colitis is a rare disorder characterized by diffuse gastrointestinal polyposis, chronic diarrhea, weight loss, and systemic manifestations such as hypoalbuminemia, alopecia, and nail dystrophy [1,2]. With fewer than 500 documented cases worldwide, its pathophysiology remains poorly understood. Diagnosis is based on clinical presentation, endoscopic findings, and histological confirmation of inflammation with eosinophilic infiltration [3,4]. 

While treatment often involves corticosteroids and nutritional supplementation, the disease has a high mortality rate, estimated at 55% within five years of diagnosis [2,5]. We report a case of inflammatory polyposis colitis in a 79-year-old male who presented with typical symptoms and showed clinical improvement with corticosteroid therapy.

## Case presentation

Clinical presentation and history

A 79-year-old male with a medical history of hypertension, hyperlipidemia, and coronary artery disease presented to the outpatient gastroenterology clinic with complaints of persistent diarrhea, weight loss, and systemic symptoms.

The patient reported up to 20 episodes of watery, non-bloody diarrhea daily over 6-8 weeks, accompanied by a 15-pound weight loss, nausea, and anorexia. He also noted nail cracking, hair thinning, and a sensation of a lump in his throat. He denied recent travel, antibiotic use, or dietary changes. His social history included past smoking but no alcohol consumption. 

Physical examination 

The patient appeared generally well but exhibited dry skin and nail dystrophy. Abdominal examination was unremarkable, revealing no tenderness, masses, or organomegaly. Table 1 lists the findings of laboratory tests.

**Table 1:** Laboratory values

Laboratory Test	Patient Value	Reference Value
Albumin	3.4 g/dL	3.5 - 5.0 g/dL
Erythrocyte Sedimentation Rate	39 mm/hour	0 - 20 mm/hour
C-reactive Protein	22 mg/L	< 10 mg/L
Total Bilirubin	1.76 mg/dL	0.1 - 1.2 mg/dL
Stool Studies	"-" for infection	Negative

Endoscopic findings

**Figure 1:**
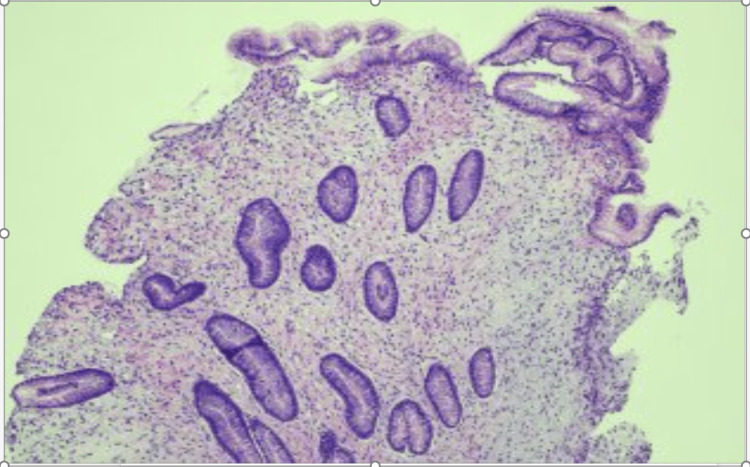
Gastric antrum on 10 x magnification depicting striking lamina propria edema with mucosal thickening and hyperplasia

Upper endoscopy and colonoscopy revealed erythematous, granular mucosa in the stomach and diffuse polypoid changes throughout the colon. Biopsies showed acute and chronic inflammation with significant eosinophilic infiltration. Histopathological findings confirmed the diagnosis of inflammatory polyposis colitis (Figure 1).

Treatment and outcome

The patient was started on oral budesonide (9 mg/day), resulting in the resolution of diarrhea within one week. He was also referred to a nutritionist to address vitamin and mineral deficiencies. Follow-up at three months showed no recurrence of symptoms.

## Discussion

Inflammatory polyposis colitis is a rare and poorly understood condition, with pathophysiological mechanisms believed to involve immune dysregulation and chronic inflammation [1,3]. Its hallmark features include diffuse gastrointestinal polyposis and systemic manifestations such as hypoalbuminemia, alopecia, and nail dystrophy [2,4].

The average age of onset is 61 years, with no significant gender predilection [2]. Chronic diarrhea and weight loss are the most common presenting symptoms, as observed in our patient. Hypoalbuminemia, present in up to 88% of cases, contributes to systemic symptoms such as edema and dermatological changes [6].

Treatment typically involves corticosteroids to reduce inflammation and modulate immune responses, though their efficacy is not well-established in controlled studies [5,7]. In severe cases, immunosuppressants such as tacrolimus or surgical interventions may be considered.

Our case demonstrates the successful use of budesonide in alleviating symptoms and highlights the importance of early diagnosis and intervention to prevent complications. 

## Conclusions

Inflammatory polyposis colitis is an exceedingly rare condition requiring a high index of suspicion for diagnosis. This case underscores the importance of corticosteroid therapy and nutritional support in management while further research is needed to elucidate its pathophysiology and optimize treatment strategies.
